# Correction objectives have higher impact than screw pattern and density on the optimal 3D correction of thoracic AIS: a biomechanical study

**DOI:** 10.1007/s43390-020-00275-2

**Published:** 2021-01-26

**Authors:** Luigi La Barbera, A. Noelle Larson, Carl-Eric Aubin

**Affiliations:** 1 Department of Mechanical Engineering, Polytechnique Montreal, Downtown Station, P.O. Box 6079, Montreal, QC H3C 3A7 Canada; 2 Research Center, Sainte-Justine University Hospital Center, 3175, Cote Sainte-Catherine Road, Montreal, QC H3T 1C5 Canada; 3 Department of Orthopedic Surgery, Mayo Clinic, Rochester, MN USA; 4 Laboratory of Biological Structure Mechanics, Department of Chemistry, Materials and Chemical Engineering, Giulio Natta, Politecnico di Milano, Piazza Leonardo da Vinci 32, 20133 Milano, Italy

**Keywords:** Adolescent idiopathic scoliosis (AIS), Correction objectives, Screw pattern, Implant density, Multibody model, Optimization, Patient-specific biomechanical modeling

## Abstract

**Study design:**

Assessment of screw pattern, implant density (ID), and optimization of 3D correction through computer-based biomechanical models.

**Objective:**

To investigate how screw pattern and ID affect intraoperative 3D correction of thoracic curves in adolescent idiopathic scoliosis, and how different correction objectives impact the optimal screw pattern.

**Summary of background data:**

Screw pattern, ID, correction objectives and surgical strategies for posterior fusion of AIS are highly variable among experienced surgeons. The “optimal” instrumentation remains not well defined.

**Methods:**

10 patient-specific multibody models of representative adolescent idiopathic scoliosis Lenke 1A cases were built and used to compare alternative virtual correction surgeries. Five screw patterns and IDs (average: 1.6 screws/instrumented level, range: 1.2–2) were simulated, considering concave rod rotation, *en bloc* derotation, and compression/distraction as primary correction maneuvers. 3D correction descriptors were quantified in the coronal, sagittal and transverse planes. An objective function weighting the contribution of intraoperative 3D correction and mobility allowed rating of the outcomes of the virtual surgeries. Based on surgeon-dependent correction objectives, the optimal result among the simulated constructs was identified.

**Results:**

Low-density (ID ≤ 1.4) constructs provided equivalent 3D correction compared to higher (ID ≥ 1.8) densities (average differences ranging between 2° and 3°). The optimal screw pattern varied from case to case, falling within the low-density screw category in 14% of considered scenarios, 73% in the mid-density (1.4 < ID < 1.8) and 13% in the high-density. The optimal screw pattern was unique in five cases; multiple optima were found in other cases depending on the considered correction objectives.

**Conclusions:**

Low-density screw patterns provided equivalent intraoperative 3D correction to higher-density patterns. Simulated surgeon’s choice of correction objectives had the greatest impact on the selection of the optimal construct for 3D correction, while screw density and ID had a limited impact.

**Level of evidence:**

N/A.

## Introduction

The treatment of adolescent idiopathic scoliosis (AIS) through posterior instrumented fusion has significantly evolved in the last decades. Since the introduction of pedicle screws, correction techniques have continuously evolved, allowing powerful control on 3D spine shape through a variable number of anchoring points [1]. Screw pattern and implant density (ID, defined as the number of implants per vertebra over the instrumented spine segment) may be expected to affect the selection of surgical maneuvers to achieve adequate 3D correction. Although these aspects are certainly related, the high inter-surgeons variability (ID range 1–2) indicates that there is no consensus [2–13]. Considering that high implant density has been associated with increased surgery time, blood loss, complications and costs [14, 15], using fewer implants may be beneficial. While previous retrospective studies are underpowered to confirm the clinical advantages of decreased implant density [15–17], a recent randomized controlled trial (RCT) by the Minimize Implants Maximize Outcomes (MIMO) Study Group demonstrated that equivalent correction in the coronal plane could be achieved using lower IDs [18].

3D correction objectives are defined as the choice to target one or more specific descriptors of spinal deformity on different anatomical planes (i.e., Cobb angle, transverse plane vertebral rotation, thoracic kyphosis) using specific maneuvers. Surgeon preference regarding correction objectives are believed to be responsible for the high variability among surgeons and may affect the final outcome [2, 19, 20]. The lack of standardization regarding surgical techniques affected by variable surgeon experience, knowledge and perspective of the complex biomechanics of 3D spine deformity correction is an additional issue [21].

Patient-specific computer-based surgical simulations are a valuable tool to virtually test the impact of a variety of screw patterns, densities and techniques on 3D correction in a finely controlled environment excluding confounding factors [2, 21–24]. Using such tools, Wang et al. did not find significant differences of correction with different implant densities (ID) following simulated concave rod rotation and compression/distraction correction maneuvers [24]. Martino et al. reported improved transverse plane correction using a maximal density (ID = 2) construct compared to a very low-density one (ID = 1), following concave rod rotation and *en bloc* derotation [26]. Le Naveaux et al. found that ID at the concave side significantly affected coronal correction, while the overall ID was weakly associated to transverse plane correction [2]; they concluded that low-density patterns, with implant mainly placed on the concave side, could result in a comparable simulated deformity correction as higher densities. Delikaris et al. also simulated an additional step of compression/distraction, reporting a strong correlation between ID in the apical region and transverse plane correction, but equivalent results in the coronal and sagittal planes [22].

Early studies on the optimization of personalized surgery planning for AIS patients [27–29], only analyzed a few cases with hybrid (screws/hooks) constructs with fixed implant patterns for each curve and simplified surgical maneuvers (i.e., concave rod rotation) without considering more recent derotation techniques.

The aims of the present comparative computational study are: (i) to investigate how screw pattern and ID affect intraoperative 3D correction in thoracic scoliosis following concave rod rotation, *en bloc* derotation and compression/distraction maneuvers, (ii) to study how correction objectives affect the optimal screw pattern.

Our first hypothesis is that low (ID ≤ 1.4 screws/level) vs. high (ID ≥ 1.8) density screw patterns would not significantly affect the correction in the three anatomical planes. The second hypothesis is that surgeon’s choice of specific correction objectives would greatly influence the choice of the optimal screw pattern.

## Materials and methods

### Cases’ selection

10 representative thoracic AIS cases (Lenke 1A) with main thoracic (MT) Cobb angle of 63° ± 6°, a T4–T12 thoracic kyphosis (TK) of 30° ± 20°, an apical vertebral rotation (AVR) of 17° ± 7° and curve flexibility of 40% ± 17% were analyzed (Table 1).

**Table 1:** Demographic data and geometric indices of the presenting deformities. Flexibility was calculated from side bending radiographs

Patient	#1	#2	#3	#4	#5	#6	#7	#8	#9	#10	Avg ± SD
Sex	Female	Female	Male	Female	Female	Female	Female	Female	Female	Female	
Age (years)	12	18	16	14	17	15	16	17	16	14	16 ± 2
Height (cm)	151	156	166	167	156	166	166	166	154	144	159 ± 2
Weight (kg)	39	53	64	57	49	73	70	55	50	42	55 ± 11
MT Cobb angle (°)	61	57	67	60	69	56	60	63	60	74	63 ± 6
End vertebrae for Cobb angle evaluation	T5-L1	T5-T12	T6-L1	T5-T12	T7-L2	T7-L1	T6-T12	T5-T12	T7-L2	T5-T12	
Curve flexibility (%)	25	37	53	74	51	28	23	21	35	50	40 ± 17
T4-T12 TK (°)	6	40	7	15	34	34	41	34	19	73	30 ± 20
AVR (°)	31	14	11	16	16	24	23	8	8	14	17 ± 7
Apex	T9	T9	T9	T9	T10	T10	T9	T9	T11	T9	

### Patient-specific biomechanical surgery model

A personalized computer biomechanical model was built for each case using MD Adams 2018 Multibody Dynamics Simulation Solution (MSC Software, Santa Ana, CA) following an established workflow from calibrated pre-operative biplanar radiographs [2, 21, 22, 24, 26]. Vertebrae were modeled as rigid bodies connected using flexible intervertebral disc and ligament structures, whose stiffness matrix globally was calibrated based on pre-operative clinical side bending radiographs [30].

Homogeneous conditions were analyzed for all curves, rather than simulating the actual surgery; therefore, the simulated posterior fixation included ten vertebrae with uniaxial screws and cobalt-chromium rods with a 5.5 mm diameter. Rod shapes were reconstructed from available postoperative radiographs. Based on the literature, five alternative screw patterns with a variety of implant densities (independent variables) were considered for each curve (Fig. 1): alternate (“A”, ID = 1.2) [3, 5, 10], periapical dropout (“PAD”, ID = 1.4) [6], convex alternate (“CA”, ID = 1.6) [5, 7, 10], convex periapical dropout (“CPAD”, ID = 1.7) [6] and a full bilateral (“B”, ID = 2) instrumentation [8, 9].

**Fig. 1:**
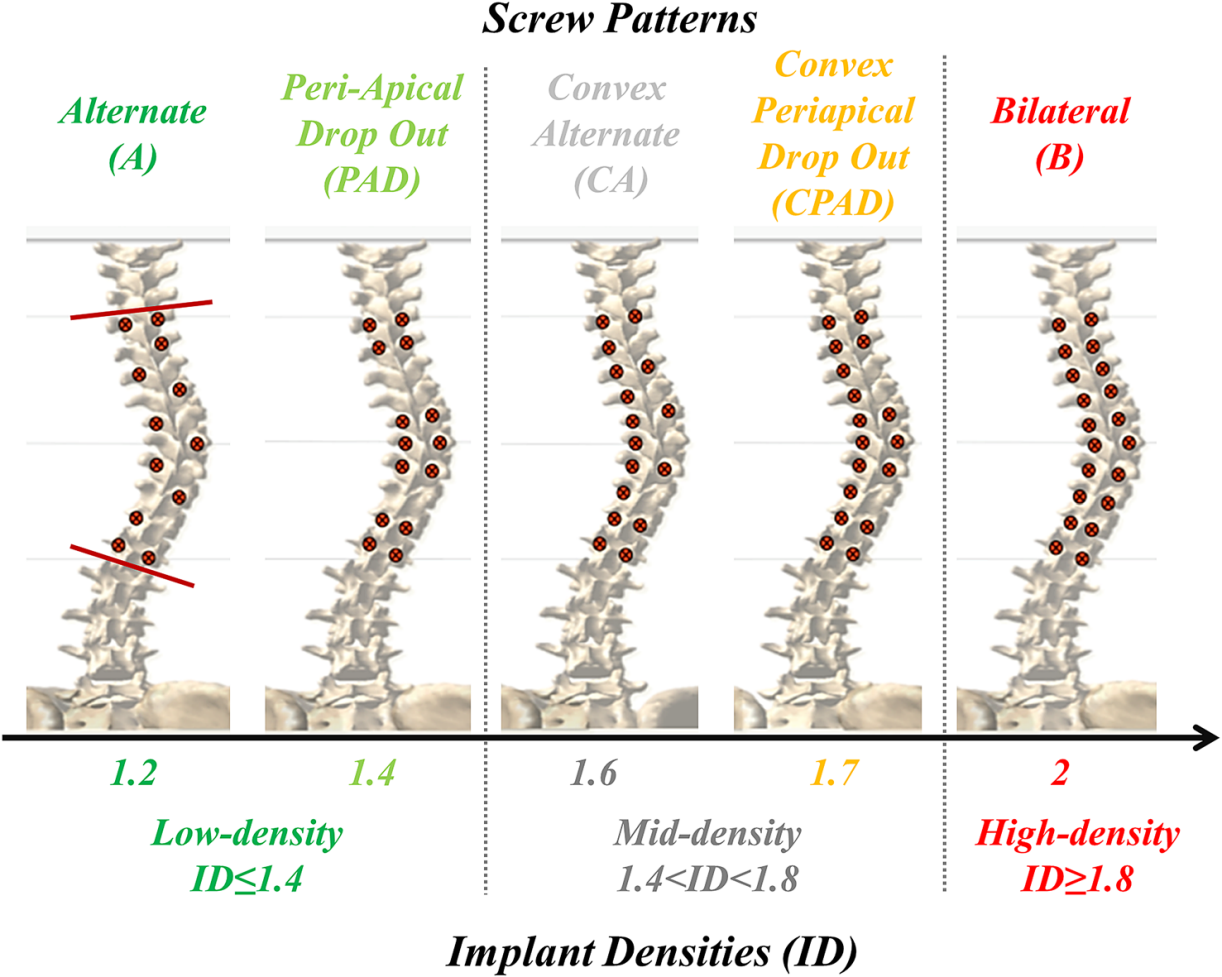
Simulated screw (red dots) patterns and corresponding implant densities (ID). Low- and high-density thresholds of 1.4 and 1.8 were defined according to the MIMO Study Group [18]

The simulated correction maneuvers were homogeneous for all cases, to isolate the effects of the tested independent variables:

- concave rod positioning and screw engagement,
- concave rod rotation (torque-controlled) [22, 26],
- distal set-screw tightening,
- *en bloc* derotation (torque-controlled) applied bilaterally on the apical and periapical screws [22, 26],
- apical screws tightening,
- convex rod positioning and attachment,
- compression/distraction,
- final screw tightening.

### Deformity correction: 3D descriptors and statistical analysis

To assess the effect of screw pattern and ID (independent variables) on the intraoperative 3D correction of the instrumented MT curves, dependent descriptors in the coronal, sagittal and transverse planes were calculated and compared to the corresponding pre-operative condition, as the baseline reference, for each curve: constrained MT Cobb angle, T4-T12 TK and AVR.

After a preliminary Kolmogorov–Smirnov normality check, a two-tailed paired Student *T*-test allowed to compare the simulated 3D descriptors to the reference ones (significance level *p* = 0.05). The same simulated procedure allowed detecting differences solely due to screw patterns and ID.

To evaluate the sensitivity of the predicted 3D descriptors on screw pattern/ID, their variability was quantified reporting average, standard deviation and overall range (maximum to minimum).

### Optimization strategy

A simple objective function was used to rate the outcome of the virtual surgery weighting the contribution of the predicted 3D descriptors of the scoliotic curve and its residual mobility [27, 28]:

ϕ =  $${{W}_{1}\cdot \left(\frac{MT Cobb}{{MT Cobb}_{0}}\right)}^{2}+{{W}_{2}\cdot \left(\frac{TK- {TK}_{n}}{{TK}_{0}- {TK}_{n}}\right)}^{2}+{W}_{3}\cdot {\left(\frac{MT AVR}{{MT AVR}_{0}}\right)}^{2}+{W}_{4}\cdot {\left(\frac{{N}_{Fused}}{{N}_{0}}\right)}^{2}$$

where the postoperative predicted $MT Cobb$ angle, $TK$ and $MT AVR$ were normalized to the corresponding preoperative measurements (indicated with the underscript “0”). As for the TK, the formula compared the simulated post-op. and the presenting values with the normo-kyphotic range: ${TK}_{n}$ = 20° if $TK$ < 20°, $TK$ = ${TK}_{n}$ if 20° ≤  $TK$ ≤ 40° [31, 32], ${TK}_{n}$ = 40° if $TK$ > 40°. The mobility term described the post-op. number of fused vertebrae (${N}_{\mathrm{Fused}}$) compared to those initially available (${N}_{0}$ = 17 thoracic and lumbar vertebrae); this term was here assumed constant with ${N}_{\mathrm{Fused}}$ = 10 for all the simulated curves and patterns.

Surgeon’s preference for one or more of the above-mentioned descriptors, specified by the weighting terms (${W}_{i}$), was derived from a previous survey on experienced surgeons of the Scoliosis Research Society and Spinal Deformity Study Group (Table 2) [20, 28]. This resulted in 550 possible combinations (10 curves, 5 screw patterns/ID, 11 correction objectives).

**Table 2:** Weights (%) assigned by 11 surgeons (S1 to S11) to each term of the objective function expressing their importance for an optimal 3D correction [20, 28]

		Correction Objectives										
		S1	S2	S3	S4	S5	S6	S7	S8	S9	S10	S11
Coronal plane	${W}_{1}$	30	50	30	45	30	20	60	30	25	50	30
Sagittal plane	${W}_{2}$	30	20	30	45	30	50	30	30	10	20	10
Transverse plane	${W}_{3}$	20	10	20	10	20	20	10	20	25	20	40
Mobility	${W}_{4}$	20	20	20	0	20	10	0	20	40	10	20

Ideally, *ϕ* = *1* would indicate no correction nor residual mobility (total fusion, ${N}_{\mathrm{Fused}}={N}_{0}$) compared to pre-op.; *ϕ* =  *0* would indicate a perfect correction with preservation of mobility (${N}_{\mathrm{Fused}}$ = *0*). In general, *ϕ* <  *1* would indicate an improvement in 3D correction, with a reduction of mobility (1 ≤  ${N}_{\mathrm{Fused}}$ < ${N}_{0}$). For a specific set of correction objectives, the minimization of the objective function allowed to determine the optimal screw pattern/ID maximally correcting the deformity in the three planes for of each case, while preserving its mobility.

To evaluate the sensitivity of the output of the objective function on the correction objectives, its variability was quantified for each case, both reporting average ± standard deviation and the range (maximum-minimum). Moreover, the percentage contribution of each term of the objective function was quantified. The frequency distribution of the optimal screw patterns according to the tested correction objectives was calculated for each case.

### Data analysis

To quantify the sensitivity of the output of the objective function due to the specific curve (i.e., patient), the correction objectives and the screw pattern/ID, a factorial ANOVA was performed in Matlab (significance level *α* = 0.05).

## Results

### 3D descriptors for deformity correction

The predicted MT Cobb significantly decreased after instrumentation (*p* < 0.05), from 63% (“A” construct, ID = 1.2) up to 67% (“B”, ID = 2) of the presenting value. TK did not show significant differences compared to the presenting curve (3–6% differences, *p* > 0.81): TK increased of about 12° for hypo-kyphotic curves (TK < 20°; cases #1, #3, #4), it decreased of 10° − 22° for hyper-kyphotic curves (TK > 40; case #10), while it did not change for the normo-kyphotic ones. AVR significantly decreased after the simulated instrumentation (*p* < 0.05), improving with ID from 69% (“A”, ID = 1.2) up to 83% (“B”, ID = 2) of its presenting value.

No significant difference was detected for any 3D correction parameter between all tested screw patterns and ID (*p* > 0.16). Screw pattern had a limited effect on simulated MT Cobb, TK and AVR, which varied in average of 3.1° (± 1.4°), 2.1° (± 2.0°) and 2.8° (± 1.3°), respectively: the maximum variability of most 3D descriptors were ≤ 4.2°, with the only exception of case #6, where it reached 6.6° and 7.4°, respectively, for Cobb and TK (Table 3).

**Table 3:** Simulated post-op. values of 3D descriptors (MT Cobb, TK and MT AVR) for each of ten thoracic cases and for each simulated screw pattern

Patient	3D descriptor (°)	Screw pattern / ID					Avg ± SD	[min; max]	max–min
		A / 1.2	PAD / 1.4	CA / 1.6	CPAD /1.7	B / 2			
#1	MT Cobb	24	24	23	22	22	23 ± 1	[22; 24]	1.9
	TK	17	17	18	18	18	18 ± 0	[17; 18]	1.0
	AVR	1	1	1	1	1	1 ± 0	[1; 1]	0.4
#2	MT Cobb	22	22	19	19	20	20 ± 2	[19; 22]	3.8*
	TK	33	31	32	32	32	32 ± 0	[31; 33]	1.3
	AVR	− 3	1	− 1	0	0	− 1 ± 1	[− 3; 1]	3.8*
#3	MT Cobb	18	17	16	16	15	16 ± 1	[15; 18]	2.7
	TK	19	19	20	19	19	19 ± 0	[19; 20]	1.3
	AVR	− 5	− 1	− 2	− 1	− 1	− 2 ± 2	[− 5; − 1]	3.9*
#4	MT Cobb	19	18	17	17	17	18 ± 1	[17; 19]	2.7
	TK	18	15	17	17	17	17 ± 1	[15; 18]	3.2*
	AVR	− 11	− 10	− 11	− 10	− 11	− 11 ± 1	[− 11; − 10]	1.3
#5	MT Cobb	28	29	27	26	27	27 ± 1	[26; 29]	2.2
	TK	35	37	35	36	36	36 ± 1	[35; 37]	1.8
	AVR	− 11	− 13	− 11	− 13	− 12	− 12 ± 1	[− 13; − 11]	1.8
#6	MT Cobb	24	22	18	18	17	20 ± 3	[17; 24]	6.6**
	TK	39	41	34	36	36	37 ± 3	[34; 41]	7.4**
	AVR	5	6	7	8	8	7 ± 1	[5; 8]	3.5*
#7	MT Cobb	27	25	25	25	25	26 ± 1	[25; 27]	2.5
	TK	43	43	42	42	42	42 ± 1	[42; 43]	1.3
	AVR	− 14	− 11	− 11	− 11	− 11	− 12 ± 1	[− 14; − 11]	2.7
#8	MT Cobb	27	25	24	24	24	25 ± 1	[24; 27]	3.2*
	TK	35	35	34	34	34	35 ± 0	[34; 35]	1.1
	AVR	− 1	3	2	3	3	2 ± 2	[− 1; 3]	4.2*
#9	MT Cobb	23	21	20	19	20	20 ± 1	[19; 23]	3.3*
	TK	30	31	30	30	30	30 ± 0	[30; 31]	0.9
	AVR	− 6	− 5	− 4	− 4	− 4	− 5 ± 1	[− 6; − 4]	2.5
#10	MT Cobb	19	18	18	18	21	19 ± 1	[18; 21]	2.2
	TK	51	51	50	50	50	50 ± 1	[50; 51]	1.9
	AVR	− 6	− 2	− 3	− 2	− 2	− 3 ± 2	[− 6; − 2]	3.8*

Their variability is expressed as average (Avg) ± standard deviation (SD) with range (Max–min). The double asterisk ("**") indicates the maximum variability of 3D descriptors, while a single asterisk ("*") indicates when the variability is greater than the average value calculated over all ten cases

### Variability of the terms of the objective function

The output of each term of the objective function depended on screw pattern and correction objectives (tested independent variables), with an average value of 0.1 (± 0.0), 0.4 (± 0.9), 0.1 (± 0.1), and 0.1 (± 0.0) for the coronal, sagittal, transverse planes and for mobility (Table 4). The maximum variability was 0.1 for the coronal term (case #7), 3.0 for the sagittal term (case #7) and 0.2 for the transverse term (case #9). *ϕ* had an average overall variability of 0.5 (± 0.9); only cases #4 and #7 presented a higher variability (Table 4).

**Table 4:** Variability of each term of the objective function, expressed as average ± SD with range (max–min), due to a variation in correction objectives and screw pattern/ID for each curve (55 possible combinations: 5 screw patterns/implant densities and 11 correction objectives). The percentage contribution to the overall output of the objective function is also reported

Patient	Terms of the objective function	Avg ± SD	[min; max]	Max–min	Contribution to the objective function (%)	
					Avg ± SD	[min; max]
#1	Coronal plane	0.1 ± 0.0	[0.0; 0.1]	0.2*	**47** ± 22	[20; 90]
	Sagittal plane	0.0 ± 0.0	[0.0; 0.0]	0.0	8 ± 6	[2; 19]
	Transverse plane	0.0 ± 0.0	[0.0; 0.0]	0.0	0 ± 0	[0; 0]
	Mobility	0.1 ± 0.0	[0.0; 0.1]	0.1	44 ± 25	[0; 78]
#2	Coronal plane	0.1 ± 0.0	[0.0; 0.1]	0.1*	**49** ± 27	[19; 99]
	Sagittal plane	0.0 ± 0.0	[0.0; 0.0]	0.0	0 ± 0	[0; 0]
	Transverse plane	0.0 ± 0.0	[0.0; 0.0]	0.0	2 ± 1	[1; 4]
	Mobility	0.1 ± 0.0	[0.0; 0.1]	0.1	44 ± 25	[0; 78]
#3	Coronal plane	0.0 ± 0.0	[0.0; 0.0]	0.0	33 ± 27	[9; 86]
	Sagittal plane	0.0 ± 0.0	[0.0; 0.0]	0.0	1 ± 1	[0; 4]
	Transverse plane	0.0 ± 0.0	[0.0; 0.1]	0.1	11 ± 4	[5; 18]
	Mobility	0.1 ± 0.0	[0.0; 0.1]	0.1	**55** ± 29	[0; 84]
#4	Coronal plane	0.0 ± 0.0	[0.0; 0.1]	0.1	10 ± 5	[4; 20]
	Sagittal plane	0.2 ± 0.2	[0.0; 0.7]	0.7*	**47** ± 18	[17; 75]
	Transverse plane	0.1 ± 0.0	[0.0; 0.2]	0.2*	26 ± 11	[13; 53]
	Mobility	0.1 ± 0.0	[0.0; 0.1]	0.1	17 ± 12	[0; 43]
#5	Coronal plane	0.1 ± 0.0	[0.0; 0.1]	0.1*	29 ± 17	[12; 63]
	Sagittal plane	0.0 ± 0.0	[0.0; 0.0]	0.0	0 ± 0	[0; 0]
	Transverse plane	0.1 ± 0.1	[0.1; 0.3]	0.2*	**48** ± 11	[27; 66]
	Mobility	0.1 ± 0.0	[0.0; 0.1]	0.1	23 ± 14	[0; 44]
#6	Coronal plane	0.1 ± 0.0	[0.0; 0.1]	0.1*	42 ± 24	[16; 88]
	Sagittal plane	0.0 ± 0.0	[0.0; 0.0]	0.0	1 ± 1	[0; 4]
	Transverse plane	0.0 ± 0.0	[0.0; 0.1]	0.0	14 ± 5	[6; 25]
	Mobility	0.1 ± 0.0	[0.0; 0.1]	0.1	**42** ± 23	[0; 72]
#7	Coronal plane	0.1 ± 0.0	[0.0; 0.1]	0.1**	6 ± 33	[2; 10]
	Sagittal plane	1.1 ± 0.7	[0.2; 3.2]	3.0**	**83** ± 11	[61; 94]
	Transverse plane	0.1 ± 0.0	[0.0; 0.1]	0.1*	5 ± 4	[1; 16]
	Mobility	0.1 ± 0.0	[0.0; 0.1]	0.1	6 ± 6	[0; 22]
#8	Coronal plane	0.1 ± 0.0	[0.0; 0.1]	0.1*	**46** ± 24	[19; 91]
	Sagittal plane	0.0 ± 0.0	[0.0; 0.0]	0.0	0 ± 0	[0; 0]
	Transverse plane	0.0 ± 0.0	[0.0; 0.1]	0.1	14 ± 5	[6; 25]
	Mobility	0.1 ± 0.0	[0.0; 0.1]	0.1	39 ± 22	[0; 69]
#9	Coronal plane	0.0 ± 0.0	[0.0; 0.1]	0.1	29 ± 18	[11; 65]
	Sagittal plane	0.0 ± 0.0	[0.0; 0.0]	0.0	0 ± 0	[0; 0]
	Transverse plane	0.1 ± 0.1	[0.0; 0.3]	0.2**	**42** ± 10	[22; 59]
	Mobility	0.1 ± 0.0	[0.0; 0.1]	0.1	30 ± 17	[0; 53]
#10	Coronal plane	0.0 ± 0.0	[0.0; 0.1]	0.0	22 ± 13	[9; 53]
	Sagittal plane	0.0 ± 0.0	[0.0; 0.1]	0.1	26 ± 16	[6; 56]
	Transverse plane	0.0 ± 0.0	[0.0; 0.1]	0.1	9 ± 3	[4; 18]
	Mobility	0.1 ± 0.0	[0.0; 0.1]	0.1	**43** ± 24	[0; 78]

The double asterisk ("**") indicates the maximum variability of each term of the objective function, while a single asterisk ("*") indicates when the variability is greater than the average value calculated over all ten curves. The percentage contribution to the overall output of the objective function is expressed as Avg ± SD with range

The percentage contribution of each term to the overall output varied from case to case. Considering the average of all screw patterns and correction objectives, the predominant component was the coronal for cases #1, #2 and #8, the sagittal for cases #4 and #7, the transverse for cases #5 and #9, and mobility for cases #3, #6 and #10 (Table 4).

### Optimal screw pattern (and ID)—General trends

Among all the curves and the tested correction objectives, “CPAD” pattern was optimal in 39% of analyzed scenarios (43/110), followed by “CA” in 35% (38/110), “A” in 14% (15/110) and “B” in 13% (14/110). When grouping by ID, low-density patterns resulted optimal in 14% of analyzed scenarios (or 15/110), mid-density patterns in 74% (or 81/110) and high-density in 13% (14/110).

### Optimal screw pattern (and ID)—Specific trends

The optimal pattern was unique for 50% of the patients. One specific mid-density pattern was optimal in three cases: “CA” for cases #5 and #7, “CPAD” for cases #2 and #9. The “B” high-density pattern was optimal for case #3. Multiple optimal patterns were found for the remaining patients, depending on the tested correction objectives (Table 5). The optimal pattern was within the mid-density range for cases #1 and #10. The optimum could span from low- to mid-density patterns both for cases #4 (“A”: 8/11, “CPAD”: 3/11) and #8 (“A”: 7/11, “CPAD”: 3/11, “CA”: 1/11). In case #6, the optimal pattern could span from mid- (“CA”: 8/11) to high-density (“B”: 3/11).

**Table 5:** Overall variability of the objective function and corresponding optimal screw patterns for each curve. When the optimum is unique, the corresponding screw pattern is bolded; when more than one screw pattern resulted to be optimal, their frequency distribution over the 11 tested correction objectives is reported as a percentage

Patient	Avg ± SD	[min; max]	Max–min	Optimal screw patterns
#1	0.1 ± 0.0	[0.1; 0.2]	0.1	CA: 55%; CPAD:45%
#2	0.1 ± 0.0	[0.1; 0.2]	0.1	**CPAD**
#3	0.1 ± 0.0	[0.0; 0.2]	0.2	**B**
#4	0.3 ± 0.1	[0.2; 0.8]	0.7**	A: 73%; CPAD: 27%
#5	0.2 ± 0.1	[0.1; 0.4]	0.3	**CA**
#6	0.1 ± 0.0	[0.1; 0.2]	0.1	CA: 73%; B: 27%
#7	1.3 ± 0.7	[0.4; 3.3]	2.9	**CA**
#8	0.1 ± 0.0	[0.1; 0.2]	0.1**	A: 64%; CPAD: 27%; CA: 9%
#9	0.2 ± 0.1	[0.1; 0.4]	0.3	**CPAD**
#10	0.1 ± 0.0	[0.1; 0.2]	0.1	CPAD: 91%; CA: 9%

The double asterisk ("**") indicates the maximum variability of objective function output, while a single asterisk ("*") indicates when the variability is greater than the average value calculated over all ten cases

### Data analysis

The ANOVA indicated that the output of the objective function was significantly affected by the specific curve (97%, *p* < 0.05), rather than by the correction objectives and the screw pattern/ID. When repeating the ANOVA for each term of the objective function, the specific curve could explain 36% (*p* < 0.05), 96% (*p* < 0.05) and 84% (*p* < 0.05) of the variability of the coronal, the sagittal and transverse terms, respectively; while the correction objectives could explain 56% (*p* < 0.05, higher than patient effect) of the coronal term and 7% (*p* < 0.05) of the transverse one; and the screw pattern/ID only explained an 8% (*p* < 0.05) of the coronal term.

## Discussion

The present biomechanical study systematically compared five similar screw patterns and IDs (independent parameters), widely accepted among experienced spine surgeons [3, 5–11], while controlling the preparation technique (no osteotomies or ligament release), the instrumented levels and correction maneuvers (confounding parameters here constant). Conversely, traditional clinical studies included inhomogeneous hybrid patterns, various implant densities, fused levels and correction techniques [11, 15–17].

The first hypothesis that low- and high-density screw patterns would provide equivalent 3D correction was confirmed, as we did not find statistically significant differences on coronal, sagittal and transverse plane correction. This generally agrees with previous biomechanical studies on thoracic AIS curves describing concave rod rotation, *en bloc* derotation and eventual compression/distraction [2, 21, 22, 26]. Compared to our study (ID range 1.2–2; 10 fused levels), they considered also lower, but today less common, IDs (range 0.73–2; fused levels 10–11) [2, 26], reporting significant effects/correlations with ID only in few specific cases.

Our results are consistent with the MIMO prospective RCT on Lenke 1A curves, reporting equivalent coronal correction of MT Cobb (range 63%–67%) and unchanged TK using low-/high-densities [18], however, correction maneuvers and implant patterns were not documented. When considering concave rod rotation and eventual compression/distraction, published retrospective studies reported equivalent coronal (63%–76%) [4, 5, 10, 33–35], sagittal (TK change − 6°–14°) [4, 5, 10, 21, 33–35] and transverse plane correction (AVR change: 1°) [33] mixing various patterns and arbitrary definitions of low/high IDs. When considering our same set of maneuvers, an equivalent coronal correction (80%–84%) was again reported for relatively low IDs (1.1 vs. 1.3)[36]. Although we did not, they reported unsatisfactory sagittal restoration with lower IDs and small (5.5 mm) rod diameters, while a better TK with higher ID and bigger size (6.35 mm) rods [36]. Our same qualitative trend on TK, with a slight decrease in hyper-kyphotic curves and significant increase in hypo-kyphotic ones, have been reported [4, 21]. Although variations in the surgical technique and implants (i.e., rods contour/size) may explain some relative differences among studies [2], our analysis confirmed that screw pattern and ID play a minor contribution on correction, when pooling the correction maneuvers, the number of fused levels and using CoCr 5.5 mm rods. Based on preliminary simulations [41], we also ensured that the effect of rod diameter on 3D correction was even lower than the effect of screw pattern and ID herein presented. Considering the impact of using high-density constructs on increased surgery time, blood loss, complications and costs [14, 15, 34], using fewer implants to achieve adequate correction could be considered.

This paper provides an important novel contribution in that it identifies the optimal screw patterns and implant densities linked to specific correction objectives. Previous studies focused only on hybrid constructs, fixed implant pattern for each curve without modern derotation techniques [27–29]. The current study demonstrates that the choice of the same correction objectives would not result in the same optimal screw pattern in a representative cohort of ten thoracic curves, confirming our second hypothesis and supporting three ideas. Firstly, each curve is unique due to a combination of patient-specific variables (i.e., curve flexibility/extension, apex location), stressing on the need for a personalized approach to support the decision-making process for pre-operative surgical planning. Unfortunately, when comparing patients’ characteristics (i.e., curve flexibility, pre-op. Cobb, pre-op. thoracic kyphosis, pre-op. apical vertebral rotations, % coronal correction, variation in thoracic kyphosis, % transverse correction) with the characteristics of the screw patterns and IDs identified as optimal, we did not notice anything relevant. The ANOVA indicated that the objective function was significantly more affected by the specific curve (i.e., patient) rather than by the correction objectives and the screw patterns or IDs; therefore, we suspect that a higher number of curves would be needed to possibly assess how patients’ characteristics are associated with the optimal instrumentation strategy. As second, the identification of the optimal screw pattern/ID depends on subjective correction objectives with multiple optimal pattern being equally justified for the same curve. As the spectrum of surgical choices is wide [11, 12, 16, 17, 19, 20], only a systematic individualized approach like the one here proposed could help rationalizing the decision-making process and reduce its variability. As third, correction objectives have higher impact than the tested independent parameters on the optimization result. For instance, a too low weight factor may hide the effect of a clinically relevant (> 5°) variation of a specific deformity indices in a specific plane; vice versa, a too high weight factor may overweight the effect of a not clinically relevant (< 5°) variation in other plane. To avoid these situations and preserve useful information, the weight factors’ range could be limited depending on the variability of each corresponding 3D descriptor. This is expected to be clearer as multiple independent parameters (e.g., rods tracing and stiffness, the number of fused vertebrae, correction maneuvers, spine flexibility…), which are expected to have a much higher impact on 3D correction than screw pattern and ID alone, are included in the modelling.

The tested correction objectives were extracted from a survey at a period where surgeons were rating more the coronal plane (Table 2) [20, 28]. Nowadays, bi-planar low-dose radiography and transverse plane maneuvers are more established, while the attention on sagittal plane and balance is increasing [37]; therefore, correction perspectives could be different today.

Assumptions in the modeling may set some limitations on the results of this study. To limit the number of tested independent variables, the same number of fused levels, fixed screw patterns/ID and correction techniques were simulated for all cases. While, in reality, surgeons may adjust the surgical technique depending on the available anchoring points, curve flexibility and span a wider range of possibilities [2, 11, 26]. Additional factors, such the anchor type [27, 29, 38], rod characteristics (i.e., contour, material, diameter) [27, 39, 40], other surgical maneuvers (i.e., segmental derotation, in situ rod bending) [21], might affect the correction in the three anatomical planes. Moreover, changing the number of fused levels (upper/lower instrumented vertebrae) [27–29] would impact spine mobility in the objective function. Only a more general simulation study accounting for all the desired instrumentation parameters and their interactions, could establish how they would ensure an optimal correction [41].

The objective function here considered was limited to one key metric per plane describing the intraoperative condition, but it may be further expanded to include other spine descriptor, as well as other non-geometrical parameters (i.e., loads at implants/bone interface, loads on pedicle screws and spinal rods, loads on the anterior spine), which have already been reported in dedicated studies [2, 22, 25, 26]. The presented approach has the potential to be used in future complementary study to evaluate other research questions, such as the risks of failure due to postoperative functional loads arising during the everyday life activities. The developed optimization approach could be applied to any other Lenke curve type. Although these aspects would merit further investigation, the effect of our study assumptions on the final conclusions could be, nevertheless, considered as limited, given its comparative nature and its focus on screw pattern and ID.

To conclude, this biomechanical comparative study demonstrates that screw patterns with low (ID ≤ 1.4 screws/level) and high (ID ≥ 1.8) implant densities provide equivalent intraoperative correction in the three anatomical planes in a representative cohort of ten thoracic AIS curves. Moreover, the identification of the optimal screw pattern for every curve depends on surgeons’ preference regarding specific correction objectives.

The proposed patient-specific approach represents a promising tool to assess and optimize the surgery planning of complex spinal deformities including surgeon-dependent correction objectives.
